# Novel Biodegradable Polymeric Microparticles Facilitate Scarless Wound Healing by Promoting Re-epithelialization and Inhibiting Fibrosis

**DOI:** 10.3389/fimmu.2018.02851

**Published:** 2018-12-04

**Authors:** Maxim A. Nosenko, Anastasia M. Moysenovich, Ruslan V. Zvartsev, Anastasia Y. Arkhipova, Anastasia S. Zhdanova, Igor I. Agapov, Tamara V. Vasilieva, Vladimir G. Bogush, Vladimir G. Debabov, Sergei A. Nedospasov, Mikhail M. Moisenovich, Marina S. Drutskaya

**Affiliations:** ^1^Engelhardt Institute of Molecular Biology, Russian Academy of Sciences, Moscow, Russia; ^2^Department of Biology, Lomonosov Moscow State University, Moscow, Russia; ^3^Moscow Regional Research and Clinical Institute (“MONIKI”), Moscow, Russia; ^4^V. I. Shumakov National Medical Research Center of Transplantology and Artificial Organs, Moscow, Russia; ^5^State Research Institute for Genetics and Selection of Industrial Microorganisms of National Research Center “Kurchatov Institute”, Moscow, Russia

**Keywords:** tissue regeneration, polymer particles, fibroin, spidroin, scar resolution, TNF, IL-6

## Abstract

Despite decades of research, the goal of achieving scarless wound healing remains elusive. One of the approaches, treatment with polymeric microcarriers, was shown to promote tissue regeneration in various *in vitro* models of wound healing. The *in vivo* effects of such an approach are attributed to transferred cells with polymeric microparticles functioning merely as inert scaffolds. We aimed to establish a bioactive biopolymer carrier that would promote would healing and inhibit scar formation in the murine model of deep skin wounds. Here we characterize two candidate types of microparticles based on fibroin/gelatin or spidroin and show that both types increase re-epithelialization rate and inhibit scar formation during skin wound healing. Interestingly, the effects of these microparticles on inflammatory gene expression and cytokine production by macrophages, fibroblasts, and keratinocytes are distinct. Both types of microparticles, as well as their soluble derivatives, fibroin and spidroin, significantly reduced the expression of profibrotic factors *Fgf2* and *Ctgf* in mouse embryonic fibroblasts. However, only fibroin/gelatin microparticles induced transient inflammatory gene expression and cytokine production leading to an influx of inflammatory Ly6C+ myeloid cells to the injection site. The ability of microparticle carriers of equal proregenerative potential to induce inflammatory response may allow their subsequent adaptation to treatment of wounds with different bioburden and fibrotic content.

## Introduction

Skin wound healing is a complex process that involves initial coagulation and rapid myeloid cells infiltration resulting in inflammatory response followed by proliferative and re-epithelialization phases (1). The balance between these processes determines the dynamics of regeneration, as well as the outcome of wound healing, in particular, fibrosis, scar formation, and efficient restoration of skin appendages (2). The inflammatory phase that appears to be a trigger and a crucial regulator of skin regeneration features activation of multiple proinflammatory cytokines (3, 4). Among them TNF and IL-6 demonstrate the highest impact on wound healing, however, their role in this process remains controversial. TNF signaling was initially shown to be detrimental for skin regeneration as TNFRp55 (TNFRI) deficiency led to accelerated wound healing and increased re-epithelialization in mice (5). At the same time, it was associated with accumulation of collagen at the wound bed, which could be indicative of fibrosis (6) and scar formation. Further studies demonstrated that TNF is important for inducing ILC3 migration to the wound site and for triggering the regeneration process (7). In particular, administration of recombinant TNF to skin wounds accelerated healing, while local TNF blockade resulted in delayed regeneration. Importantly, TNF produced by myeloid cells was shown to be crucial for the quality of skin regeneration, since TNF-deficient mice, as well as myeloid cell-restricted TNF knock-out mice, revealed disrupted wound-induced hair neogenesis (WIHN) (8). The role of IL-6, another proinflammatory cytokine, in skin wound healing was also investigated using reverse genetics. Interestingly, IL-6-deficient, but not IL-6R-deficient animals were characterized by delayed regeneration (9–11) and impaired hair neogenesis (12–14). Since STAT3 activation was shown to be crucial for wound healing (15, 16), a possibility of other IL-6 family cytokines involvement in the skin regeneration process should be considered.

At the same time, when the key inflammatory players in skin wound healing were elucidated, there was a push to derive micro- and nanoparticles (MPs and NPs) that can be used as carriers for introduction of certain cell types or drugs into the injured skin to facilitate rapid and scarless wound healing (17–19). While these preliminary results supported the continued exploration of biomaterial-derived particles for tissue repair, the *in vivo* proregenerative potential of MPs with no regards to administered cells or growth factors was not investigated. In this work we aimed to clarify (a) the contribution to the wound repair of the transferred microparticles; (b) the impact of these biopolymeric scaffolds on the proinflammatory mediators of wound repair; and (c) the extent of similarities and differences in mechanism of action of different biomaterial-derived scaffolds.

To answer these questions, we have evaluated two types of previously described silk-based MPs, generated from fibroin/gelatin (20, 21) scaffolds or spidroin (22–24) hydrogels and characterized their regenerative and inflammatory potential *in vivo* in the model of murine deep skin wounds, as well as direct effects on inflammatory and profibrotic genes expression in several cell types involved in skin regeneration *in vitro*. We have demonstrated that, while possessing similar proregenerative potential, these vehicles are remarkably different in their interaction with the host immune system. This unexpected result suggests that eventual adaptation of a biopolymer carrier to the type of injury to be healed will allow “point-of-care” optimization of properties of the medicine delivered by such a carrier.

## Materials and Methods

### Mice

C57Bl/6 mice were bred at the Pushchino Animal Breeding Facility (Branch of the Shemyakin and Ovchinnikov Institute of Bioorganic Chemistry, Russian Academy of Sciences), housed under specific pathogen-free conditions with 12 h light/dark cycle at room temperature. Animals with age of 7–9 weeks were used for the experiments with MPs. All manipulations with animals were carried out in accordance with recommendations in the Guide for the Care and use of Laboratory Animals (NRC 2011), the European Convention for the protection of vertebrate animals used for experimental and other scientific purposes, Council of Europe (ETS 123), and “The Guidelines for Manipulations with Experimental Animals” (the decree of the Presidium of the Russian Academy of Sciences of April 02, 1980, no. 12000-496). All animal procedures were approved by the Scientific Council of the Engelhardt Institute of Molecular Biology.

### Generation of Microparticles

Fibroin-gelatin microparticles were generated by cryodestruction of fibroin composite sponge scaffolds substituted with 30% gelatin, as previously described (20). The resulting microparticles were successively passed through the laboratory strainers with pore size of 500, 250, and then 100 μm. The target fraction was represented by microparticles that passed through the pore size of 500 and 250 μm, but did not pass through the pore size of 100 μm. Microparticles from recombinant spidroin rS1/9 were prepared by physical crushing of hydrogels prepared from a 3% solution of rS1/9 in a 10% solution of lithium chloride in 90% formic acid with subsequent dialysis against distilled water (25) and have size range between 100 and 300 μm. Both types of MPs were stored in 70% ethanol and were washed 3 times in PBS prior to use. For *in vivo* experiments wounds were generated and then microparticles were s.c. injected as 50% suspension in PBS in three injections per skin site with 20 μl each injection (in total of 60 μl per site), for *in vitro* experiments 50% suspension of microparticles was added to an equal volume of cell suspension. Soluble fibroin (sF) and spidroin (sSp) were used as controls *in vitro* at concentration of 1 mg/ml in cell culture medium.

### Surface Morphology Analysis

The surface structure of microparticles was analyzed by confocal laser scanning microscopy (CLSM). The polymer was conjugated with TRITC according to the following procedure: microparticles were soaked in a solution containing 2.5 mg/mL of TRITC in 0.1 M bicarbonate buffer, the reaction was terminated by immersing the microparticles in 0.1 M TRITC solution for 20 min. The microparticles were then transferred to a potassium phosphate buffer solution for 15 min to remove the residual TRITC. To obtain a 3D reconstruction, a series of optical sections were created using an Eclipse Ti-E microscope with an A1 (Nikon Corporation, Japan) confocal module and a CFI Plan Apo VC 20 × /0.75 objective. Preparation of microparticles for SEM was performed according to standard protocol. Briefly, MPs were fixed overnight using 2.5% glutaraldehyde in 0.1 M cacodylate buffer at + 4°C. The samples were then washed three times in 0.1 M cacodylate buffer with pH = 7.2 for 5 min, followed by dehydration in series of ethanol solutions with increasing concentrations and acetone (Chemmed, Russia). After critical point drying using Hitachi critical point dryer HCP-2 (Hitachi, Ltd., Japan) MP's were metallized with 20 nm thick platinum layer using Ion Coater IB3 (Eiko Engineering Co., Japan). The resulting samples were analyzed with Camscan S2 microscope (Cambridge Instruments, UK) at 10 nm resolution and operating voltage−20 kV. Images were obtained using MicroCapture software (SMA, Russia).

### Cell Cultures

Primary mouse embryonic fibroblasts (MEF) were generated as previously described (26). Primary murine bone marrow-derived macrophages (BMDM) were generated by flushing the femurs and culturing bone marrow cells for 10 days according to the standard protocol (27, 28) in DMEM supplemented with 30% conditioned medium from L929 cells (a source of M-CSF) and 20% horse serum (Biological Industries, Kibbutz, Israel, lot No. 1630708). Primary mouse keratinocytes were isolated and cultured according to previously published protocol (29). At least two independent isolates for all primary cell cultures were prepared. For the experiments, MEF and BMDM were cultured on plastic dishes in DMEM (Gibco), supplemented with 10% fetal bovine serum (Gibco), and penicillin/streptamicin/L-glutamin (Gibco). Keratinocytes were cultured in DMEM/Ham's F12 (3.5: 1.1) medium supplemented with 0.05 mM Ca^2+^, 10% FCS (FCS Gold, PAA), 0.18 mM adenine (Sigma Aldrich), 0.5 μg/ml Hydrocortisone (Sigma Aldrich), 5 μg/ml insulin (Invitrogen), 10^−10^ M cholera toxin (Sigma Aldrich), 10 ng/ml EGF, 2 mM glutamine, 1 mM pyruvate. In order to determine the mRNA levels of cytokine and other inflammatory genes, 4 × 10^5^ cells per well were seeded on fibroin/gelatin or spidroin particles (100 μl of 50% suspension per well) in 24-well plate and incubated at 37°C, 5% CO_2_ for 6 and 24 h. To assess cytokine production, supernatants were collected 24 h after culturing cells in the presence of microparticles. Cell cultures without microparticles were used as a control. To measure the adhesion capacity of cells toward F/G or Sp MPs, 1 × 10^6^ cells were incubated with 250 μl of 50% suspension of MPs for 1 h at 37°C, 5% CO_2_. Suspensions were then filtered through 70 μm cell strainer (Miltenyi Biotec) to separate the unattached cells. Cells in flow-through were then counted and the resulting adhesion efficiency was calculated as (N_cells initial_ – N_cells in flow−through_)/N_cells initial_*100%.

### Immunofluorescence of Cells on Microparticles

Immunofluorescence was used to visualize MEF grown on microparticles for 7 days, as well as to analyze immune cell infiltration of microparticles *in vivo* 24 h after subcutaneous injection. In both cases, MPs were fixed with 4% paraformaldehyde and treated with 0.1% Triton X-100 in PBS for 10 min. To detect actin cytoskeleton, Alexa488-conjugated phalloidin was used. To detect Ly6C^+^ inflammatory cells, anti-Ly6C antibody, conjugated with PE-Cy7, was used (Table 1). Cell nuclei were visualized with Hoechst 33342. The images were obtained using an Eclipse Ti-E microscope with an A1 (Nikon Corporation, Japan) confocal module and a CFI Plan Apo VC 20 × /0.75 objective.

**Table 1:** Antibodies for flow cytometry analysis.

**Marker**	**Clone**	**Color**	**Dilution**	**Supplier**
Viability dye	–	eFluor710	1:3,200	ThermoFisher
CD45	30-F11	PerCP-Cy5.5	1:800	
CD11b	M1/70	APC	1:400	
Ly6G	1A8-Ly6g	FITC	1:400	
Ly6C	HK1.4	PE-Cy7	1:1,600	

### Cell Viability Assay

Viability of keratinocytes, MEF and BMDM after 1, 3, and 7 days of *in vitro* culture on fibroin/gelatin and recombinant spidroin MPs was determined with LIVE/DEAD™ Viability/Cytotoxicity Kit for mammalian cells (ThermoFisher), according to manufacturer's instructions. Stained cells were observed using an Eclipse Ti-E microscope with an A1 (Nikon Corporation, Japan) confocal module and a CFI Plan Apo VC 20 × /0.75 objective.

### MTT-Test

Cell cultures were established on fibroin/gelatin and recombinant spidroin MPs as described above. After 1, 3, and 7 days, 200 μl of MTT solution (5 mg/ml in PBS) were added and incubated at 37°C for 4 h. The cell suspension with the microparticles was collected and centrifuged at 14,500 g, the precipitate was dissolved in DMSO, and colorimetric measurements were carried out at 540 nm.

### Deep Skin Wounds

Deep skin wounds were introduced according to previously published protocol (21). Briefly, mice were anesthetized with i.p. injection of 100 μl of a mixture of Zoletil 100 (Virbac) and Rometar (Bioveta) in sterile PBS at concentration of 10 and 20%, respectively. To avoid dehydration of the retina, eye drops were constantly applied to the eye area. After disappearance of all reflexes the back skin area of mice was depilated using depilation cream (Veet) and then two full-thickness skin wounds were introduced on the back skin of mice using 4mm biopsy punchers (Medex). Mice were kept on warming pad until full recovery from the anesthetic. Day of surgery was denoted as day 0. For wound morphometry mice were photographed at days 0, 1, 7, 10, and 21 using digital camera (Nikon D810 with objective AF Micro-Nikkor ED 200 mm f/4 D IF). Each day the wound area was measured from the photographs using ImageJ software and calculated as percent of initial wound area at day 0. Scar area was measured using ImageJ software at day 21 after wounding.

### Flow Cytometry

For flow cytometry analysis skin samples 10 mm × 10 mm were excised and kept in PBS on ice. Samples were subsequently cut with scissors and digested in 1 ml of digestion medium, consisting of RPMI1640 (Gibco), 0.5 mg/ml DNase I, 1 mg/ml Collagenase D and 0.1 mg/ml Liberase TL (all enzymes from Sigma Aldrich). Digestion was performed using gentleMACS Octo Dissociator with Heaters in C-tubes (Miltenyi Biotec) for 1 h at 37°C. After digestion, cells were passed through 70 μm cell strainer (Miltenyi Biotec) and washed in 9 ml of PBS. Cells were subsequently blocked with Fcγ-blocking antibody (ThermoFisher) and stained for surface markers (Table 1). The data was acquired on BD FACS Canto II and analyzed using FlowJo. For cell number assessment, counting beads (ThermoFisher) were used according to manufacturer's instructions.

### RNA Isolation and Gene Expression Analysis

RNA from samples was isolated using TriZol (Sigma Aldrich) following manufacturer's instructions. For RNA isolation skin samples were snap-frozen in liquid nitrogen, homogenized with mortar and pestle and, subsequently, resuspended in TriZol. RNA amount was assessed using Nanodrop 1000 (ThermoFisher). RNA samples were then treated with DNaseI (ThermoFisher) and used for reverse transcription (ThermoFisher) with oligo(dT) primers following manufacturer's protocol. Generated cDNA was then used for RT-PCR on Applied Biosystems (AB7500 Real Time PCR System) using premixed RT-PCR buffer (Eurogene) and gene-specific primers (Eurogene, primer sequences are summarized in Table 2). Relative gene expression was calculated according to ΔΔCt with reference gene *Actb*.

**Table 2:** Primers for qPCR analysis.

**Gene**	**Forward 5′-3′**	**Reverse 5′-3′**	**Product length**
*Actb*	CTCCTGAGCGCAAGTACTCTGTG	TAAAACGCAGCTCAGTAACAGTCC	160
*Tnf*	TAGCCCACGTCGTAGCAAAC	ACAAGGTACAACCCATCGGC	136
*Il6*	AACCACGGCCTTCCCTACTT	TTGCCATTGCACAACTCTTTTCTC	156
*Vcam1*	GACAGCCCACTAAACGCGAA	TCCTTGGGGAAAGAGTAGATGTCC	164
*Ctgf*	TGGAGGAAAACATTAAGAAGGGCA	CACACCCCGCAGAACTTAGC	124
*Fgf2*	GGCTGCTGGCTTCTAAGTGTG	TCTGTCCAGGTCCCGTTTTGG	162
*Ccl2*	AGTTAACGCCCCACTCACCT	TTGAGCTTGGTGACAAAAACTACAG	132

### ELISA

IL-6, TNF and IL-1beta levels in cell-culture supernatants were determined using a Mouse IL-6 ELISA Ready-SET-Go, Mouse TNF alpha ELISA Ready-SET-Go, and Mouse IL-1b ELISA Ready-SET-Go kits (eBioscience, San Diego, CA, USA) according to the manufacturer's instructions.

### Histology

Five days following the induction of deep skin wounds, mice were injected with anesthesia, two skin samples 15 mm × 15 mm surrounding the injury site were excised and subsequently fixed one in 4% PFA solution and another one in Bouin's solution (saturated aqueous picric acid, formalin and glacial acetic acid in ratio 15:5:1) and a histological analysis of the tissue was carried out, as previously described (21). For re-epithelialization measurement, skin samples were stained by Mallory stain and examined using an inverted Axiovert 200 M microscope (Carl Zeiss, Germany) and an AxioCam MRC 5 camera (Carl Zeiss, Germany). Wound bed length was assessed on histological slides using ImageJ software. Infiltration of immune cells inside the implanted microparticles was analyzed by hematoxylin/eosin staining.

### Statistical Analysis

Statistical analysis was carried out using GraphPad Prism software (version 6, San Diego, CA, USA). One-way and two-way ANOVA with Holm-Sidak post-test were used for multiple pairwise comparisons. The data were obtained in at least three independent experiments and presented as the mean ± SD. *p* < 0.05 were considered to indicate statistical significance.

## Results

### Biophysical Properties of Fibroin/Gelatin and Spidroin MPs

**Figure 1:**
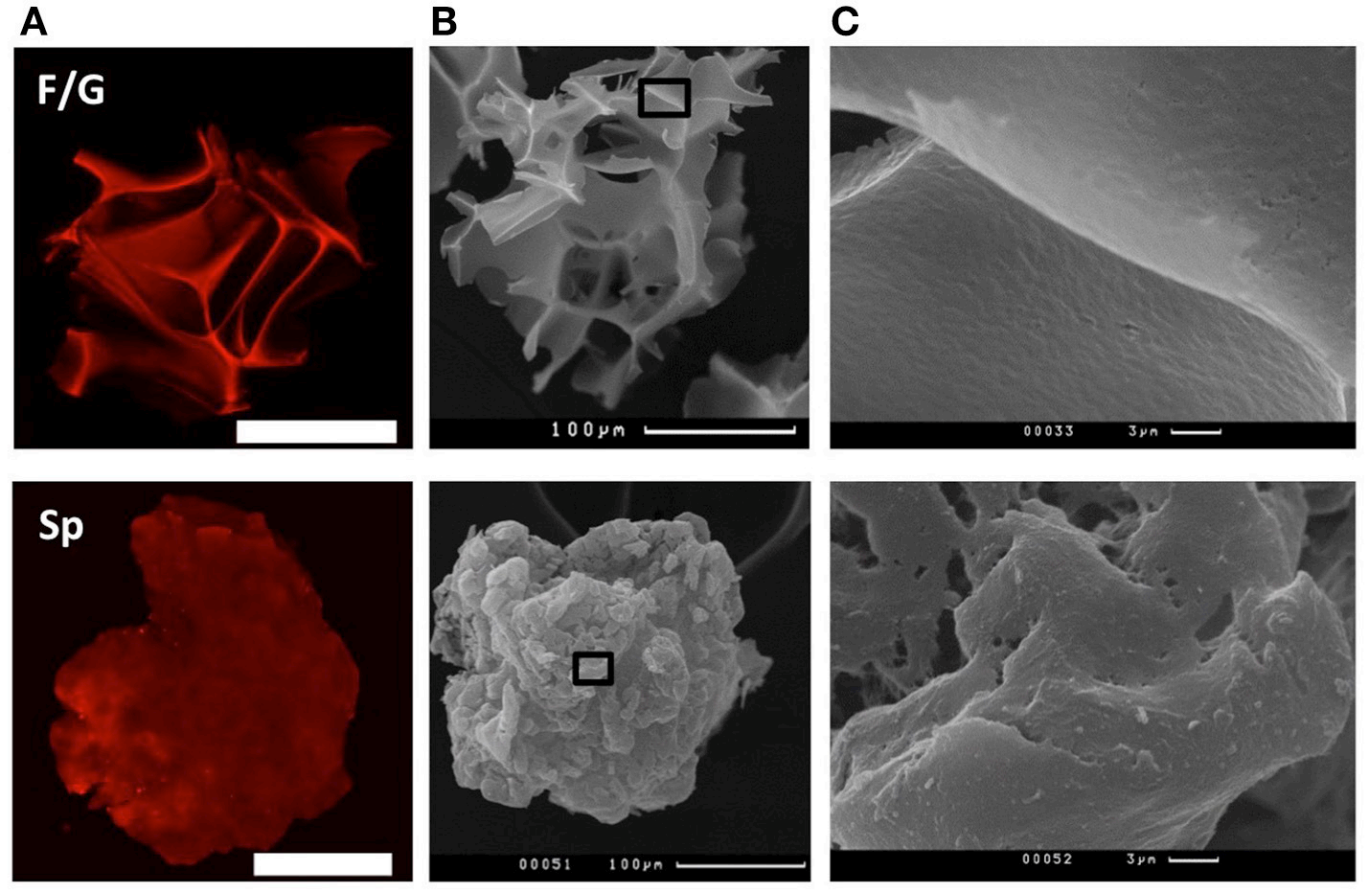
Structure of fibroin/gelatin and spidroin microparticles. **(A)** CLSM images of fibroin/gelatin or spidroin microparticles visualized by TRITC. Scale bar−100 μm. **(B)** SEM images of fibroin/gelatin or spidroin microparticles. Scale bar−100 μm. Black square indicates the region, enlarged in **(C)**.

Recombinant spidroin rS1/9-based (Sp) and fibroin/gelatin -based (F/G) microparticles (respectively, Sp MPs and F/G MPs) were used in the experiments (Figure 1). The particle size ranged from 100 to 300 μm for Sp MPs and 100–250 μm for F/G MPs. Sp MPs were obtained as a result of physical crushing of a hydrogel and represent microgel particles with a complex surface. The surface elements include nanostructures with a diameter of 100–300 nm and microstructures with a size of 10–30 μm (Figure 1C). One of the important properties of spidroin rS1/9 is a pronounced positive charge of the molecules, which is due to the presence of 29 Arg residues in the absence of negatively charged residues (theoretical pI: 10.49) (22, 30). For recombinant spidroin, the alternation of short hydrophobic and hydrophilic regions is characteristic, which allows the surface of the microgel to exhibit either hydrophobic or hydrophilic properties, depending on the environment. F/G MPs were obtained by cryodestruction of spongy scaffolds (21, 31). The resulting F/G MPs are the fragments of a scaffold with a complex surface, providing a large surface area for cell adhesion and proliferation. At physiological pH, fibroin has a negative charge (pI = 4.2), which increases the sorbtion of biologically active molecules and improves cell migration (32). To improve the adhesion of cells to the surface of F/G MPs, gelatin, a biocompatible product of partial hydrolysis of collagen containing RGD (Arg-Gly-Asp) integrin-binding amino acid sequences, was introduced into the biopolymer. Presence of gelatin resulted in a positive effect on the adhesion, proliferation, migration, and viability of various cell types (33–36).

### Fibroin/Gelatin and Spidroin MPs Promote Wound Re-epithelialization and Inhibit Scar Formation

**Figure 2:**
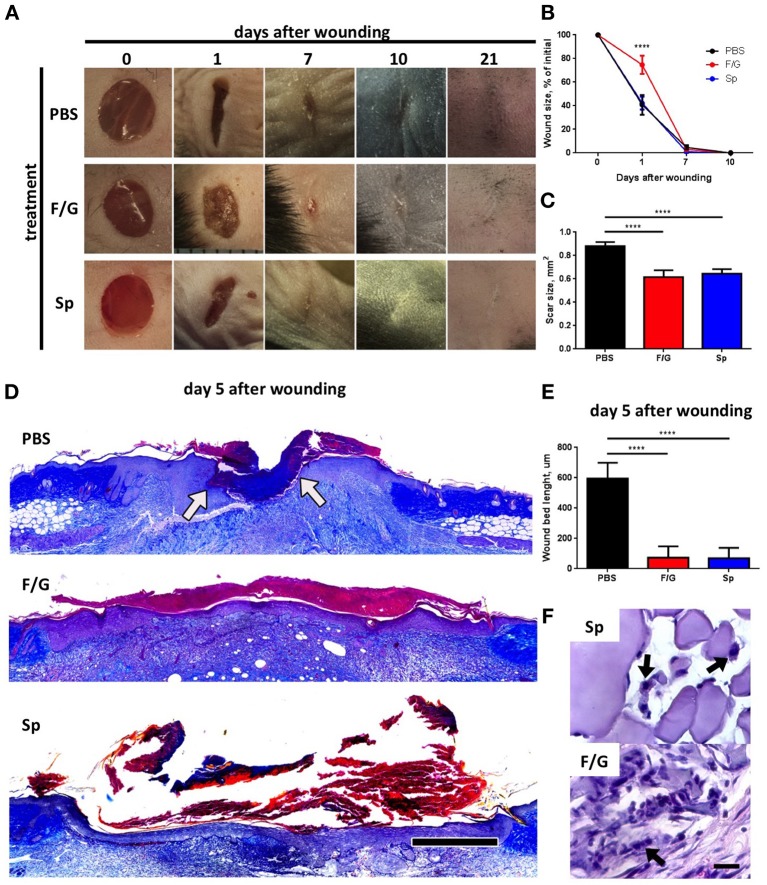
Fibroin/gelatin (F/G) and spidroin (Sp) microparticles promote wound re-epithelialization and inhibit scar formation. **(A)** Representative photographs of deep skin wounds after subcutaneous injection of fibroin/gelatin or spidroin MPs compared to control treatment (PBS). **(B)** Wound size as % of initial area at indicated timepoints calculated from wound photographs (*n* = 6). **(C)** Scar size in mm^2^ at day 21 calculated from wound photographs (*n* = 7). **(D)** Representative histological sections of wounds at day 5 stained with Mallory stain. Arrows indicate edges of wound bed. Scale bar−500 μm. **(E)** Wound bed size calculated from histology sections on day 5 after wounding (*n* = 5). **(F)** Fibroin/gelatin and spidroin MPs, revealed by H&E staining of wound samples at day 5. Arrows indicate infiltrating immune cells. Data are representative of at least three independent experiments. ****p < 0.0001.

To investigate the proregenerative potential of silk-based MPs, we first compared the ability of two types of MPs (Figure 1) to promote deep skin wound healing after subcutaneous injection. Interestingly, administration of fibroin/gelatin MPs resulted in delayed wound contraction at day 1 as compared to PBS- and spidroin-treated wounds (Figures 2A,B). However, at day 7 no difference in wound size was found (Figure 2B, day 7) and complete wound closure was observed at day 10 in all three groups (Figure 2B, day 10). Since skin fibrosis is an important indicator of the quality of wound healing, we measured the scar surface forming at the wound site on day 21 following wound induction (Figure 2B, day 21) and observed a significant reduction in scar formation after treatment with MPs as compared to the control group treated with PBS (Figure 2C). Furthermore, analysis of histological sections of wounded tissue revealed significant improvement in re-epithelialization rate after treatment with both types of MPs as compared to the control (Figures 2D,E). Interestingly, at day 5 following the s.c. injection of MPs, F/G ones, but not Sp MPs, were surrounded by the immune cells as revealed by histological staining (Figure 2F). In summary, both fibroin/gelatin and spidroin MPs showed comparable potential to promote skin wound re-epithelialization and prevent fibrosis.

### Only Fibroin/Gelatin MPs Induce Expression of Proinflammatory Cytokines in MEF and BMDM, While Both Fibroin/Gelatin and Spidroin MPs Downregulate Profibrotic Growth Factor Expression in MEF

**Figure 3:**
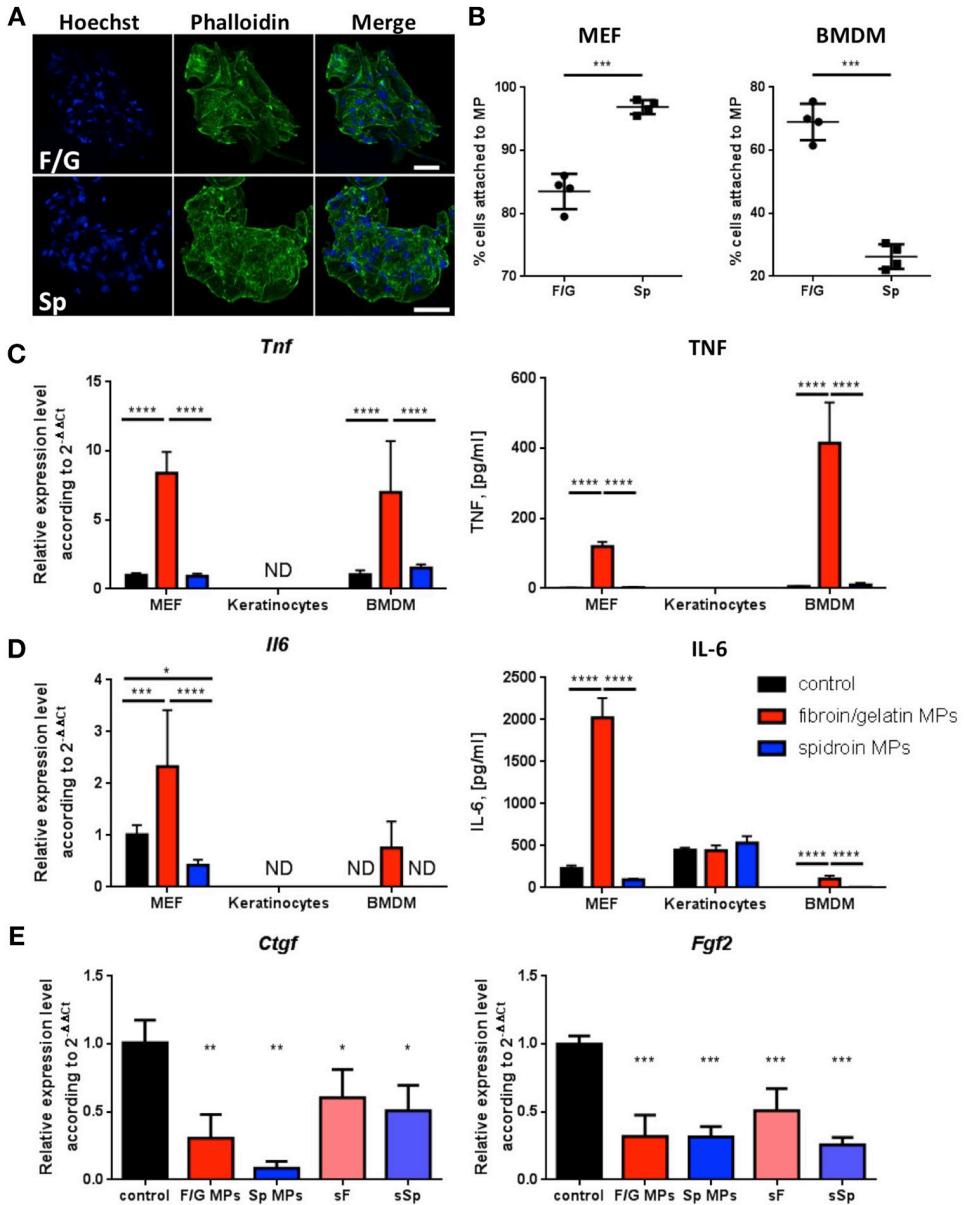
Only fibroin/gelatin MPs induce expression of proinflammatory cytokines in MEF and BMDM, while both fibroin/gelatin and spidroin MPs downregulate profibrotic growth factors expression in MEF. **(A)** MEF monolayer after 1 week in culture on fibroin-gelatin (F/G) or spidroin (Sp) microparticles. The actin cytoskeleton is stained by phalloidin-Alexa488 (green), the nuclei are stained by Hoechst 33342. Scale bar−50 μm. **(B)** Adhesion efficiency of MEF and BMDM toward fibroin/gelatin and spidroin MPs (*n* = 4). **(C,D)** Expression of TNF **(C)** and IL-6 **(D)** by three cell types cultured on fibroin/gelatin or spidroin MPs, when compared with conventional 2D cultures (control) as revealed by qPCR after 6 h (left) and by ELISA in supernatants after 24 h (right). **(E)** Expression of fibroblast growth factors genes, *Ctgf* and *Fgf2*, by MEF, cultured for 6 h on fibroin/gelatin or spidroin MPs, when compared with soluble fibroin or spidroin (sF or sSp) and conventional 2D cultures (control) as revealed by qPCR analysis (*n* = 3). Data are representative of at least three independent experiments. *p < 0.05; **p < 0.01; ***p < 0.001; ****p < 0.0001.

Since only fibroin/gelatin MPs delayed wound contraction (Figures 2B,C), we decided to further characterize *in vitro* the direct effects of both types of carriers on cells, involved in wound healing, such as fibroblasts, keratinocytes and macrophages. We established primary cultures of murine embryonic fibroblasts (MEF), keratinocytes and bone-marrow derived macrophages (BMDM) and cultured them on two types of MPs, as well as in conventional 2D culture conditions as a control. Both types of MPs supported cell growth (Figure 3A, Supplementary Figure 1A) and were associated with high viability of cells for at least 1 week of culture (data not shown). Interestingly, two types of MPs differed in their ability to promote adhesion of fibroblasts and macrophages, which represent the two main cell types interacting with MPs upon their subcutaneous injection. F/G MPs supported adhesion of BMDM, while MEF were highly adhesive to Sp MPs after 1 h of incubation (Figure 3B). To a lesser extent, keratinocytes also adhered better to Sp MPs (Supplementary Figure 1B). We next analyzed the expression of proinflammatory genes in cells cultured on two types of MPs. Surprisingly, we observed a significant induction of proinflammatory cytokines TNF and IL-6 both at mRNA and protein levels in MEF and BMDM, cultured on fibroin/gelatin but not on spidroin MPs (Figures 3C,D). Interestingly, MEF appeared to be the major source of IL-6, while BMDM produced mostly TNF in response to fibroin/gelatin MPs. However, expression of proinflammatory genes was transient and disappeared after 24 h of culture (Supplementary Figure 1D). Keratinocytes did not change expression levels of proinflammatory genes in response to either type of MPs, however, they upregulated expression of adhesion molecule *Vcam1* when cultured on spidroin MPs (Supplementary Figure 1C). In addition, we found a significant downregulation of *Ctgf* and *Fgf2* gene expression in MEF, cultured on either type of MPs as compared to the control (Figure 3E). These growth factors are known to promote fibrosis during skin wound healing (37). To distinguish between biomaterial contribution vs. the scaffold effects on the cells, we analyzed gene expression in MEF, cultured in the presence of soluble fibroin or spidroin proteins. Importantly, neither soluble fibroin nor spidroin (sF or sSp, respectively) induced the expression of proinflammatory cytokines in MEF (Supplementary Figure 1E). However, both proteins induced downregulation of *Ctgf* and *Fgf2* expression in MEF, suggesting that both fibroin and spidroin possess anti-fibrotic properties (Figure 3E). Thus, both types of MPs showed anti-fibrotic potential *in vitro*, while only fibroin/gelatin MPs induced transient inflammatory response in fibroblasts and macrophages.

### Subcutaneous Injection of Fibroin/Gelatin Microparticles Promotes Moderate Inflammation and Infiltration of Myeloid Cells to Mouse Skin *in vivo*

**Figure 4:**
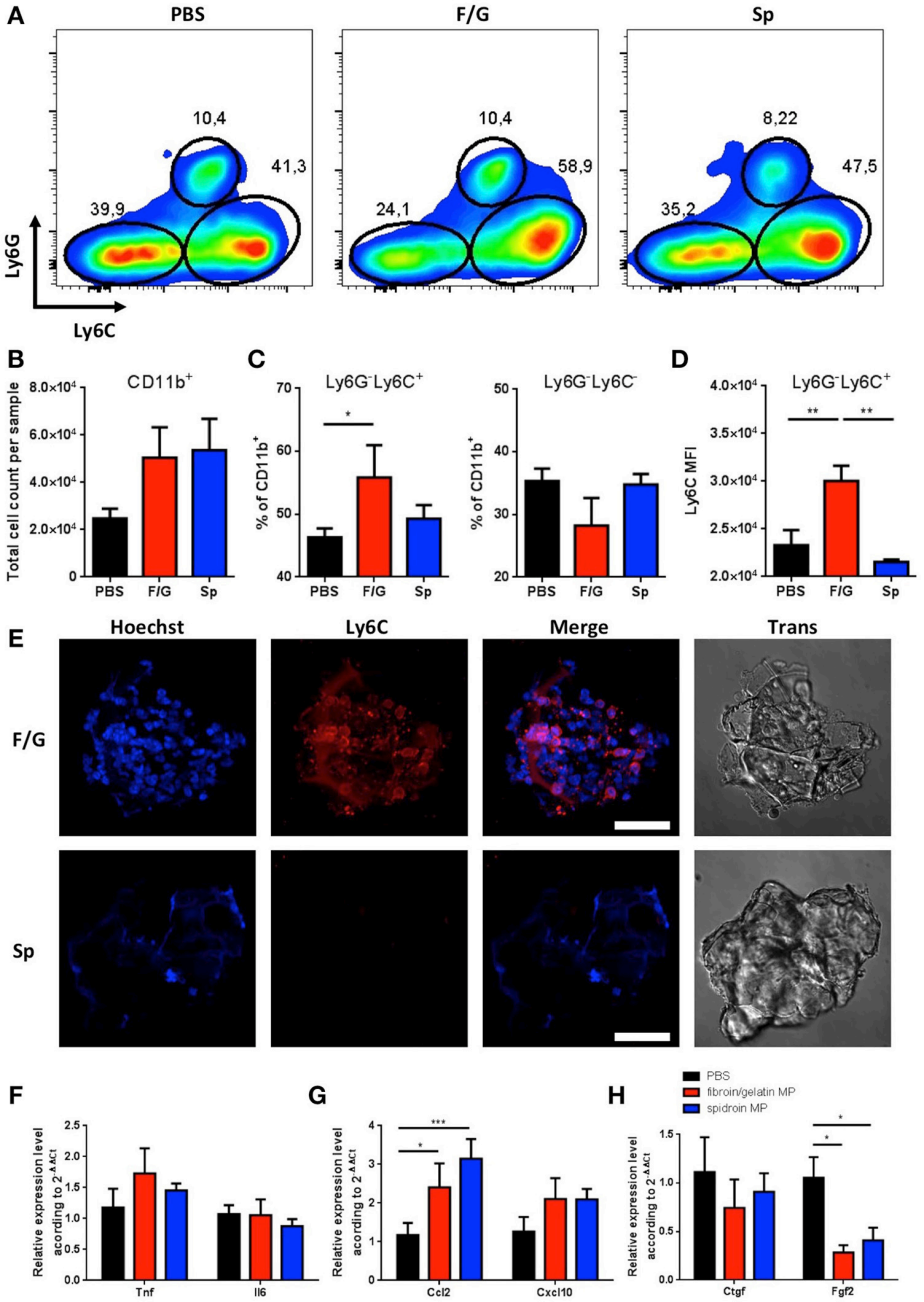
Subcutaneous injection of fibroin/gelatin and spidroin MP affects infiltration of myeloid cells and reduces expression of profibrotic growth factors. **(A)** Representative dot plots, showing expression of Ly6C and Ly6G by myeloid cells isolated from the skin 1 day after injection with PBS, fibroin/gelatin or spidroin MPs (gating strategy—Supplementary Figure 2). **(B)** Total number of CD11b^+^ myeloid cells in skin samples. **(C)** Proportion of Ly6C^hi^ and Ly6C^−^ myeloid cells in skin samples. **(D)** MFI of Ly6C staining among Ly6G^−^Ly6C^hi^ population of myeloid cells. All cells are VD^−^CD45^+^CD11b^+^. **(E)** Immunofluorescent staining of fibroin/gelatin and spidroin MPs with anti Ly6C-antibody 24 h after subcutaneous injection. Cell nuclei were stained with Hoechst 33342. **(F–H)** Expression of proinflammatory cytokines **(F)**, chemokines **(G)**, and profibrotic growth factors **(H)** in skin 1 day after injection with PBS, fibroin/gelatin or spidroin MPs. Data are representative of at least three independent experiments. *p < 0.05; **p < 0.01; ***p < 0.001.

We next investigated the short-term effects of MPs administration on skin homeostasis following subcutaneous injection. Based on our *in vitro* data (Figure 3), we expected an increase in inflammatory response and increased skin infiltration with immune cells (Supplementary Figure 2) following the injection of fibroin/gelatin MPs as compared to spidroin MPs. Interestingly, injection of either fibroin/gelatin or spidroin MPs resulted in increased skin infiltration with CD11b^+^ myeloid cells on day 1 after the injection as compared to the control group injected with PBS, although the difference did not reach the statistical significance due to high variability between the samples (Figures 4A,B). Infiltrating myeloid cells were further characterized by their proinflammatory capacity according to the expression of surface molecule Ly6C. In particular, Ly6C^hi^ cells were shown to be proinflammatory, while Ly6C^low^ cells are anti-inflammatory and proregenerative (38). In accordance with our *in vitro* data, we observed a significant increase in the percentage of highly inflammatory Ly6C^hi^ myeloid cells following the injection of fibroin/gelatin MPs as compared to spidroin MPs and the control group (Figures 4A,C). This correlated with slightly reduced proportion of non-inflammatory Ly6C^low^ cells following the injection of fibroin/gelatin MPs as compared to spidroin MPs and the control group (Figure 4C). Moreover, inflammatory Ly6C^hi^ cells, which migrated in response to fibroin/gelatin MPs, showed a significant increase in Ly6C expression as indicated by MFI (Figure 4D). To determine if these cells interact with the injected MPs, we performed an *ex vivo* immunofluorescent analysis (Figure 4E). Indeed, we observed a number of Ly6C^hi^ inflammatory cells directly attached to fibroin/gelatin, but not to spidroin MPs, in agreement with our *in vitro* data that showed increased adhesion of macrophages to fibroin/gelatin MPs (Figure 3B). Of note, we did not observe any significant changes in the number of Ly6G^+^ neutrophils in response to MPs injection (data not shown). Thus, fibroin/gelatin, but not spidroin MPs induce skin infiltration of the proinflammatory myeloid cells following subcutaneous injection.

We then analyzed the expression of major proinflammatory and proregenerative genes in the skin samples following MPs injection. In accordance with our *in vitro* data we could detect only a minor increase in the expression of *Tnf* and *Il6* genes in the skin 24 h after administration of fibroin/gelatin MPs (Figure 4F). However, injection of both types of MPs resulted in significant increase in the expression of chemokine *Ccl2* known to facilitate the immune cell infiltration during wound healing and skin inflammation (Figure 4G). This correlates with increased infiltration of myeloid cells into the skin after injection of both types of MPs as compared to control (Figure 4B). Among the genes encoding growth factors, in accordance with our *in vitro* findings, we observed a significant decrease in the expression of *Fgf2* gene in response to both types of MPs as compared to PBS-injected control group (Figure 4H). This result indicates that proregenerative potential of fibroin/gelatin and spidroin MPs may be due to their inhibitory effects on skin fibrosis.

## Discussion

It was long accepted that bioengineered MPs show their proregenerative potential mainly via providing a scaffold for cells and tissue growth, that in turn facilitate tissue regeneration (39, 40). However, the influence of biomaterials on particular cell types and their transcriptional program were largely underestimated, since it is difficult to discriminate between scaffold-based and biomaterial-based effects. Comparative studies of several types of biomaterials can provide new insights of their varying influence on cell phenotype. Indeed, our data strongly support the idea that, in addition to serving as a cell scaffold, bioengineered MPs can stimulate a distinct inflammatory gene expression pattern depending on the material used. We observed significant expression of proinflammatory genes in MEF and BMDM cultured on fibroin/gelatin, but not on spidroin MPs (Figures 3C,D), proving that fibroin/gelatin composite MPs can specifically induce transient inflammation. These data are in full agreement with recently published observations, suggesting that fibroin has proinflammatory features (41–43). However, in our work we did not detect any increase in proinflammatory signatures in cultured cells in response to soluble fibroin (Supplementary Figure 1E and data not shown), indicating that polymeric fibroin has unique cell activation properties. This is additionally supported by the published work, showing that only a certain configuration of fibroin scaffolds induces the inflammatory response in monocytes (44). Furthermore, gelatin modification, used in this work to increase biocompatibility of fibroin scaffolds, could further modulate MPs influence on cultured cells, although in previous studies we did not observe any effect of gelatin-modified fibroin scaffolds on gene expression in MEF (45).

Importantly, this proinflammatory effect is different for various cell types studied here as MEF cultured on fibroin/gelatin MPs are the main source of IL-6, BMDM mainly produce TNF, while primary keratinocytes do not overexpress either TNF or IL-6 in response to MPs (Figures 3C,D). This moderate inflammatory response results in higher infiltration of the skin by inflammatory myeloid cells after injection of fibroin/gelatin MPs (Figures 2F, 4A–E), which may be important for rapid wound healing (46). Indeed, despite delayed wound contraction (Figures 2A,B) we observed a significant increase in re-epithelialization rate of deep skin wounds after injection of fibroin/gelatin MPs (Figures 2C–E). Interestingly, although spidroin MPs did not show any proinflammatory features, they also induced overexpression of *Ccl2* (Figure 4G), resulting in increased infiltration of myeloid cells (Figure 4B), and induced rapid wound re-epithelialization upon subcutaneous injection (Figures 2D,E) with no effect on wound contraction (Figures 2A,B). This indicates that spidroin MPs possess their proregenerative features via a different mechanism. We also established that spidroin MPs strongly induced the adhesion of fibroblasts and keratinocytes *in vitro* (Figure 3B). In addition, we found an increased expression of adhesion molecule *Vcam1* in keratinocytes, cultured on spidroin MPs, as compared to fibroin/gelatin MPs (Supplementary Figure 1C), which may be beneficial for their migration during wound healing.

Further, we observed a significant anti-fibrotic effect induced by the carriers as suggested by a decrease in the expression levels of genes encoding key growth factors *Ctgf* and *Fgf2* after culturing MEF on both types of MPs (Figure 3E). Importantly, the same effect was induced by soluble fibroin and spidroin, suggesting that the choice of the biomaterial may be crucial for anti-fibrotic properties of bioengineered particles (Figure 3E). Moreover, expression of *Fgf2* was significantly downregulated after subcutaneous injection of MPs (Figure 4H). This resulted in reduced scar formation during skin regeneration, indicating of the improved wound healing quality (Figure 2C). The anti-fibrotic properties of these scaffolds are of special interest for formulation of compounds aimed at the treatment of fibrotic complications of hepatitis, autoimmune and metabolic diseases, for which the extent of fibrosis determines the rate of disease progression and eventual outcomes. In line with our initial hypothesis, we provide evidence that some effects of MP-based treatments (e.g., anti-fibrotic properties) are due to intrinsic biomaterial properties, while others (e.g., proinflammatory features of F/G MPs) seem to depend more on the scaffold structure. We believe that this aspect should be considered when evaluating novel bioengineered dressings for clinical use.

Overall, our results highlight significant opportunities in optimization of carrier vehicles previously thought to be inert and open a new avenue for rapid adaptation of therapeutic properties of drug/carrier system through reformulation techniques.

## Conclusions and Future Perspectives

We show that fibroin/gelatin and spidroin MPs have two major effects on wound healing *in vivo*: they increase re-epithelialization rate and inhibit scar formation. However, the MPs utilize different mechanisms to fulfill these tasks. Fibroin/gelatin MPs induce transient inflammatory response in MEF and BMDM *in vitro* and *in vivo* following their subcutaneous injection, resulting in the delayed wound contraction, while spidroin MPs do not exhibit any proinflammatory features, but rather support the adhesion of fibroblasts and keratinocytes *in vitro*. Both types of biomaterials downregulate expression of profibrotic factors in MEF and prevent skin fibrosis during wound healing. We have demonstrated that while possessing similar proregenerative potential, these vehicles are remarkably different in their interaction with the host's immune system. This unexpected result suggests that in the context of microparticle therapy it is possible to drastically alter its outcome by swapping a biopolymer carrier. We are hopeful that this would allow rapid optimization of microcarrier therapies to the type of the wound that needs to be healed (e.g., traumatic vs. post-operative vs. cardiometabolic, etc.) leading to a diverse set of more effective drugs and devices.

## Author Contributions

MN, AM, RZ, AA, AZ, TV, and MD performed experiments. VB, VD, and MM provided critical materials and technology. MN, MM, IA, SN, and MD designed experiments and discussed results. MN, MD, MM, and SN wrote the manuscript.

### Conflict of Interest Statement

The authors declare that the research was conducted in the absence of any commercial or financial relationships that could be construed as a potential conflict of interest.
