# Supplementary figures and images for: Novel Biodegradable Polymeric Microparticles Facilitate Scarless Wound Healing by Promoting Re-epithelialization and Inhibiting Fibrosis
Source: Front Immunol. 2018 Dec 4;9:2851. doi: 10.3389/fimmu.2018.02851 (PMC6288351; doi:10.3389/fimmu.2018.02851)

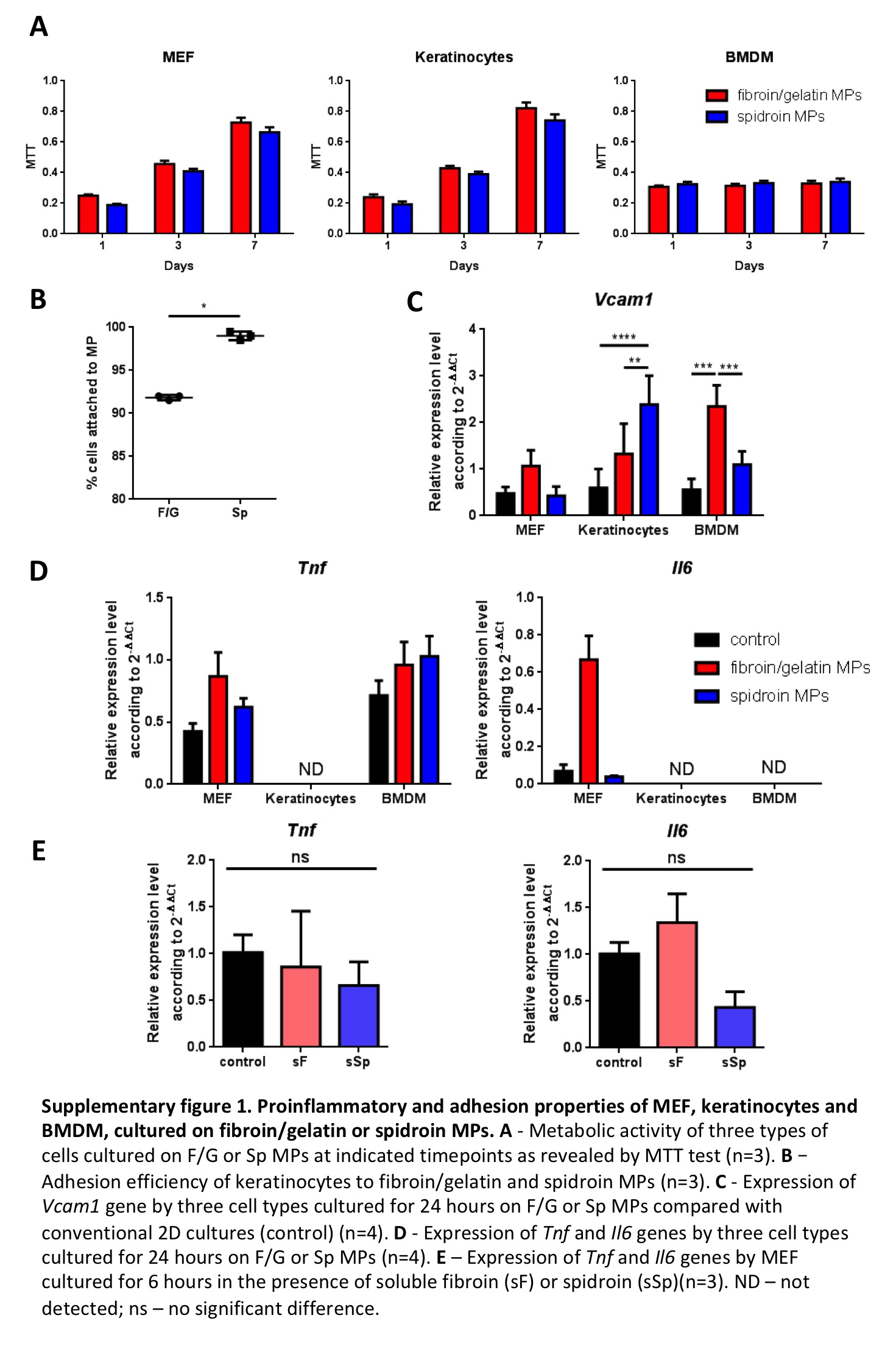

Supplement: Supplementary file 1 [file Image_1.JPEG]

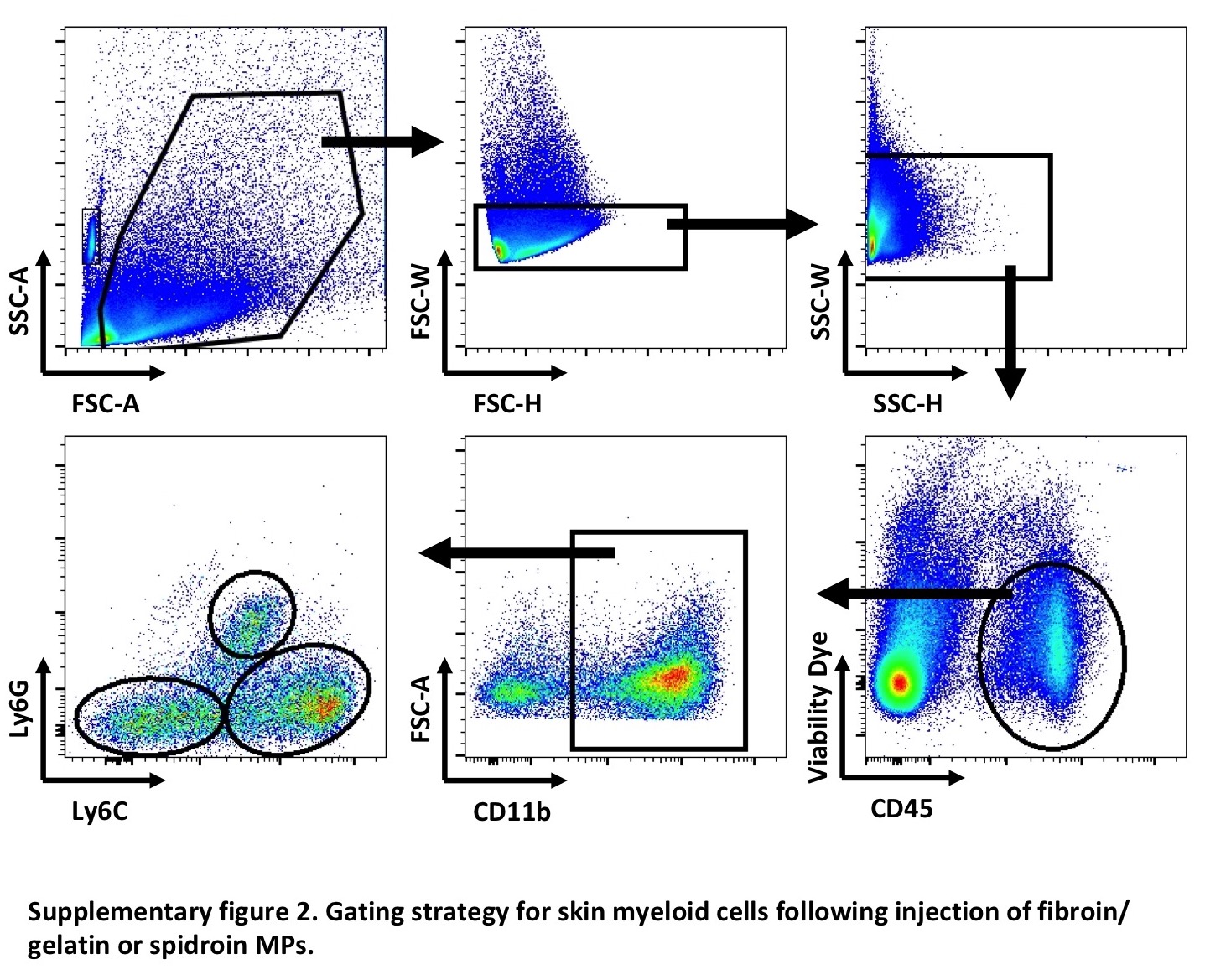

Supplement: Supplementary file 2 [file Image_2.JPEG]
